# The Current State of Intraoperative Imaging in Maxillofacial Surgery: A Systematic Review

**DOI:** 10.3390/jcm15041675

**Published:** 2026-02-23

**Authors:** Charlotte Thomas, Gary Dong, Dorien I. Schonebaum, Sanjana Challa, Alynah J. Adams, Emily Song, Fatima Arif, Jose A. Foppiani, Warren Schubert, Umar Choudry, Samuel J. Lin

**Affiliations:** 1 Division of Plastic and Reconstructive Surgery, Beth Israel Deaconess Medical Center, Harvard Medical School, Boston, MA 02215, USA; 2 Tufts University School of Medicine, Boston, MA 02111, USA; 3 Department of Plastic and Reconstructive Surgery, Amsterdam UMC, 1081 HV Amsterdam, The Netherlands; 4 Division of Plastic and Reconstructive Surgery, University of Minnesota, Minneapolis, MN 55455, USA; 5 Department of Plastic and Hand Surgery, Regions Hospital, St. Paul, MN 55130, USA

**Keywords:** maxillofacial surgery, intraoperative navigation, cone-beam computed tomography, stereotactic navigation, augmented reality

## Abstract

**Background**: In maxillofacial reconstruction, even small inaccuracies can compromise aesthetics, function, and safety. Surgeons currently rely on preoperative imaging; however, recent advances in intraoperative imaging now provide three-dimensional, real-time guidance, possibly enhancing surgical outcomes. This review evaluates the current application of intraoperative imaging in maxillary and mandibular surgery including its impact on accuracy, efficiency, and outcomes. **Methods**: Two separate systematic reviews (PROSPERO CRD420251125497, CRD420251124600), analyzing maxillary and mandibular repair were conducted through Cochrane, Medline, Embase, and Web of Science. Both reviews adhered to the PRISMA guidelines. Inclusion criteria encompassed intraoperative digital imaging or navigation in maxillary or mandibular surgery. Studies without human subjects, intraoperative imaging, or the surgery of interest were excluded. Bias was assessed with NIH Quality Assessment. **Results**: A combined total of 795 publications were screened, with 35 studies ultimately included in this review, encompassing 1643 patients. Techniques included intraoperative computed tomography (CT) (n = 12, 34.3%), stereotactic navigation (n = 16, 45.7%), augmented reality (n = 2, 5.7%), ultrasound, fluoroscopy, infrared stereoscopic and electromagnetic (n = 1, 2.9%, each). The most common indication for surgery was fracture repair. Reporting was heterogeneous, with variable metrics and reporting for accuracy, complications, and revisions. Overall, cone-beam CT (CBCT) and stereotactic navigation both demonstrated significant restoration of normal symmetry, and stereotactic navigation enabled accuracy of <2 mm. CBCT added the shortest amount of time intraoperatively, ranging from 1 to 20 min. Reporting on long-term outcomes was heterogeneous. **Conclusions**: A variety of intraoperative imaging and navigation techniques are being applied in maxillofacial surgery. However, inconsistent reporting metrics, small study size, and study feasibility-focused study design limit meaningful comparison across technologies. Rigorous prospective studies with standardized outcome measures are needed to further define their clinical value and guide adoption.

## 1. Introduction

Accurate intraoperative assessment of maxillary and mandibular alignment is critical in craniofacial surgery, as even small deviations can result in undesirable functional and aesthetic consequences [1]. Malalignment of even a few millimeters is perceptible and may negatively affect facial symmetry and aesthetic satisfaction [2]. Further, inaccuracies in maxillary positioning can lead to impaired mastication, altered speech, and malocclusion [1]. In craniofacial surgery, malalignment of orbital support structures can result in postoperative diplopia or other vision changes, and intraoperative imaging reduces these consequences. In a study of orbital floor fracture repairs, authors reported postoperative diplopia in 12 of 40 patients (30%) who underwent orbital fracture surgery with intraoperative computer tomography (CT) imaging, compared with 38 of 84 patients (45.2%) treated without intraoperative imaging [3]. These outcomes are particularly consequential in patients undergoing complex maxillofacial repairs, where precision influences functional recovery, postoperative satisfaction, and patient-reported quality of life [4]. Despite the clinical importance of accurate intraoperative assessment, however, achieving precise alignment remains a significant challenge in maxillofacial surgery. Given these functional and aesthetic consequences, reliable, accurate tools in maxillofacial surgery are essential.

Traumatic injuries constitute a significant portion of maxillofacial surgeries, and a recent Global Burden of Disease analyses reported a 19% increase in incident cases of facial fractures between 1990 and 2019, from 8.7 million to 10.7 million cases worldwide [5]. Head and neck cancer (HNC) also contributes substantially to maxillofacial reconstructive volume. Recent global burden analyses report that HNC is the seventh-most prevalent cancer worldwide, with approximately 900,000 new cases reported in 2022 [6]. Congenital craniofacial anomalies further add to maxillofacial operative demand, with cleft lip and/or palate occurring in roughly 1 per 1000 live births globally [7]. These prevalent clinical contexts underscore the need for accurate intraoperative assessment in maxillofacial surgery.

Traditionally, surgeons have relied on clinical judgement, anatomical knowledge, and preoperative imaging, such as plain radiographs and fluoroscopy, to guide repair, fundamentally limiting the intraoperative feedback accessible once in the operating room [8]. Novel tools such as patient-specific cutting guides and three-dimensional printed models were introduced to enhance preoperative planning, though they still lacked real-time intraoperative correction [9]. To address such limitations, advances such as intraoperative CT enabled efficient three-dimensional (3D) assessment of reduction and implant positioning, allowing for real-time revision [10,11]. Intraoperative imaging, CT or otherwise, refers to the acquisition of real-time images during the procedure to assess operative progress. Building on these developments, real-time 3D guidance in anatomically sensitive regions can now be provided by stereotactic navigation, image-guided tracking systems that rely in prior imaging to provide continuous spatial guidance relative to patient anatomy. Today, emerging tools such as augmented reality (AR) have enabled surgeons to overlay virtual anatomy or surgical plans directly onto the operative field [8,12]. Current studies characterize these developments and report potential gains in more accurate and reliable intraoperative revisions; however, the evidence remains heterogeneous, fragmented, and rapidly evolving.

Despite increasing interest and promise in these technologies, there is no consolidated synthesis describing the current state of intraoperative imaging in maxillofacial surgery. Prior reviews have largely focused on isolated modalities or locations, rather than providing a comprehensive assessment across technologies. Critically, advances in technology necessitate regular assessment of the tools available to surgeons. The objective of this systematic review was therefore to provide an updated summary of the current literature on intraoperative imaging in maxillary and mandibular repair and to characterize their accuracy, safety, and efficiency.

## 2. Materials and Methods

Two separate systematic reviews were planned and registered independently given the anatomic and procedural differences between maxillary and mandibular surgery. Because of substantial similarity in methodology, search design, and outcomes of interest, the authors combined the two datasets after independent screening and data extraction to provide a comprehensive synthesis of intraoperative imaging across maxillofacial procedures. The extraction for both reviews was performed separately prior to reconciliation of overlapping studies.

### 2.1. Information Sources

Through a detailed systematic literature review, articles were retrieved from Medline, Cochrane Central Register, Embase and Web of Science. The Preferred Reporting Items for Systematic Reviews and Meta-Analysis (PRISMA) guidelines were thoroughly followed (S1) [13]. This review was conducted in August and September of 2025.

### 2.2. Search Strategy

A search strategy was designed and created for both studies (S2 and S3). Both strategies were created by a professional librarian who uploaded the results into Covidence, the systematic review software used for this study. 

### 2.3. Eligibility Criteria

Study designs in both reviews included: randomized and non-randomized studies, observational studies, cohort studies, clinical trials, case reports and case series. Editorials, commentary reports, abstracts, reviews, meta-analyses, and letters to the editors were excluded. Eligible languages were limited to English. Both reviews only included studies performed with human subjects; all animal studies and basic science studies were excluded.

For the mandibular repair review, further inclusion criteria included patients with mandibular reconstruction, including but not limited to oncologic reconstruction, congenital malformation, and mandibular fractures. All included studies reported on mandibular surgical procedures utilizing intraoperative imaging or navigation. Studies were excluded if there was no report of mandibular surgery or there was no usage of intraoperative imaging. The full eligibility criteria for this review can be found at PROSPERO under protocol identification number: CRD420251124600.

The maxillary review included any study reporting patients with maxillary reconstruction. Examples of included diagnoses were oncologic reconstruction, congenital malformation, maxillary fractures including Le Fort fractures, and zygomaxillary complex (ZMC) fractures. Articles were included if there was a report of intraoperative imaging or navigation usage during maxillary surgery. Studies were excluded if there was no use of intraoperative imaging during maxillary surgery or no maxillary surgery at all. The full eligibility criteria for this review can be found at PROSPERO under protocol identification number: CRD420251125497.

### 2.4. Selection Process

For both studies, the results of the database query were uploaded to an online systematic review program, Covidence [14]. A two-stage screening process was performed by three independent reviewers (G.D., S.C., A.J.A). Stage one included title and abstract screening; after screening, a fourth, independent reviewer would resolve any conflict that occurred (C.T.). The same reviewers screened the remaining articles and performed full text analysis. After this stage, the same independent reviewer resolved any conflicts that arose.

### 2.5. Data Extraction

An extraction table was created using Google Sheets and data was extracted from all selected articles by six independent reviewers (C.T., G.D., D.S., S.C., A.J.A., F.A.). Extracted variables included: Name of first author, publication year, journal of publication, country, primary and secondary outcomes of the study, study design, number of patients, mean age, total number of patients with mandibular/maxillary repair, reason for repair, type of imaging used intraoperatively, accuracy of intraoperative imaging device, operating time, number of revision surgeries and reported complications.

### 2.6. Data Items

The primary outcomes for both reviews were similar. The main focus of this study was to analyze the current clinical use of intraoperative imaging and navigation in mandibular and maxillary repair. Other primary outcomes included safety outcomes such as complication rates and revision surgeries, as well as surgical accuracy, measured by the precision of anatomical alignment and reconstruction. Secondary outcomes included the difference in operating time and other noteworthy outcomes the articles mentioned.

### 2.7. Statistical Analysis

Although the primary outcome for most studies was similar, the reporting methods varied widely. Descriptive statistics were performed; however, further analysis was not possible due to the qualitative nature of the results.

### 2.8. NIH Quality Assessment

The National Institute of Health (NIH) has created a quality assessment tool to evaluate potential bias in the included studies. All studies were evaluated and ranked according to this tool. Studies could be ranked “poor”, “fair”, or “good” [15].

## 3. Results

**Figure 1:**
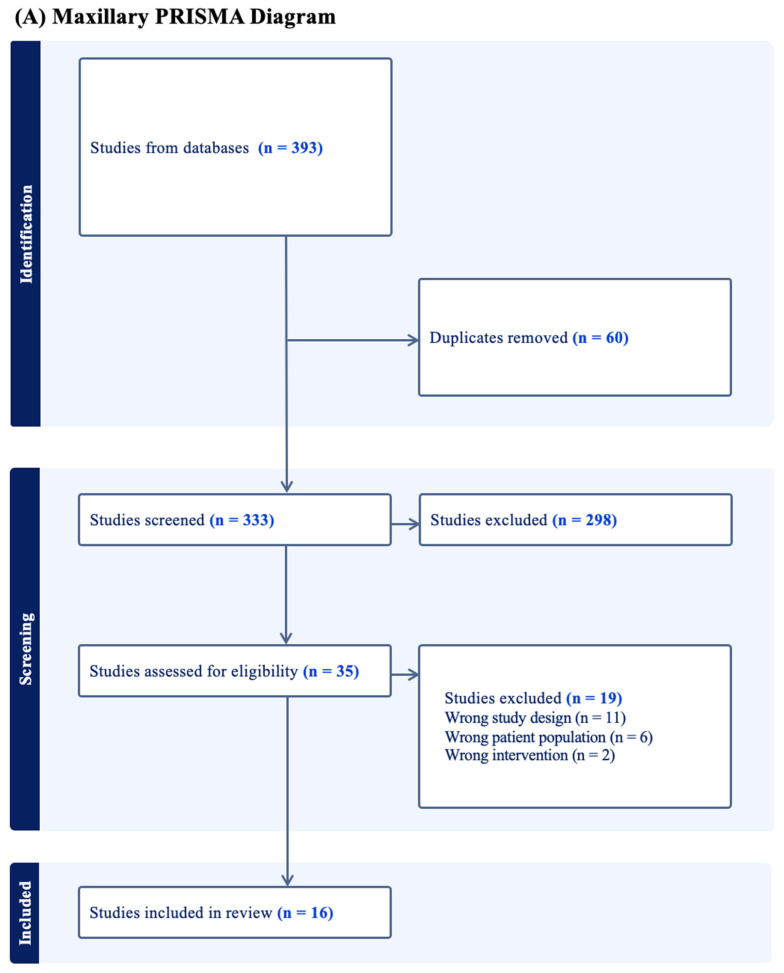

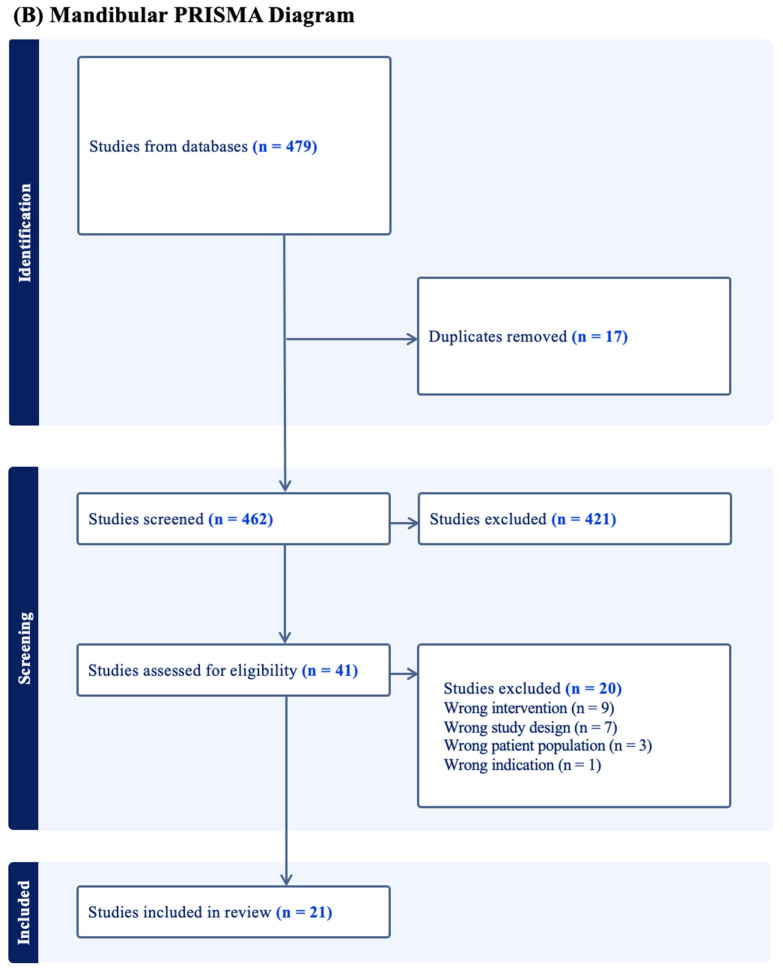
PRISMA Diagrams.

Initially, 393 maxillary studies were identified, and 16 were ultimately included for analysis [16,17,18,19,20,21,22,23,24,25,26,27,28,29,30,31]. 479 mandibular studies were identified and 21 were included for analysis (Figure 1) [23,28,32,33,34,35,36,37,38,39,40,41,42,43,44,45,46,47,48,49,50]. Two studies overlapped between both searches, yielding a total of 35 studies included in this review. Of the included studies, 14 were fracture repair (40.0%), followed by oncologic (34.3%) and mixed-indication studies (14.2%). There were 12 CT-based studies, 16 stereotactic navigation-based studies, 2 AR-based studies, and 5 unique study designs not otherwise categorized. Overall, the included studies encompass 1643 patients (Table 1 and Table 2). Eighteen studies were “good” on the NIH Quality Assessment (Table 3).

**Figure 2:**
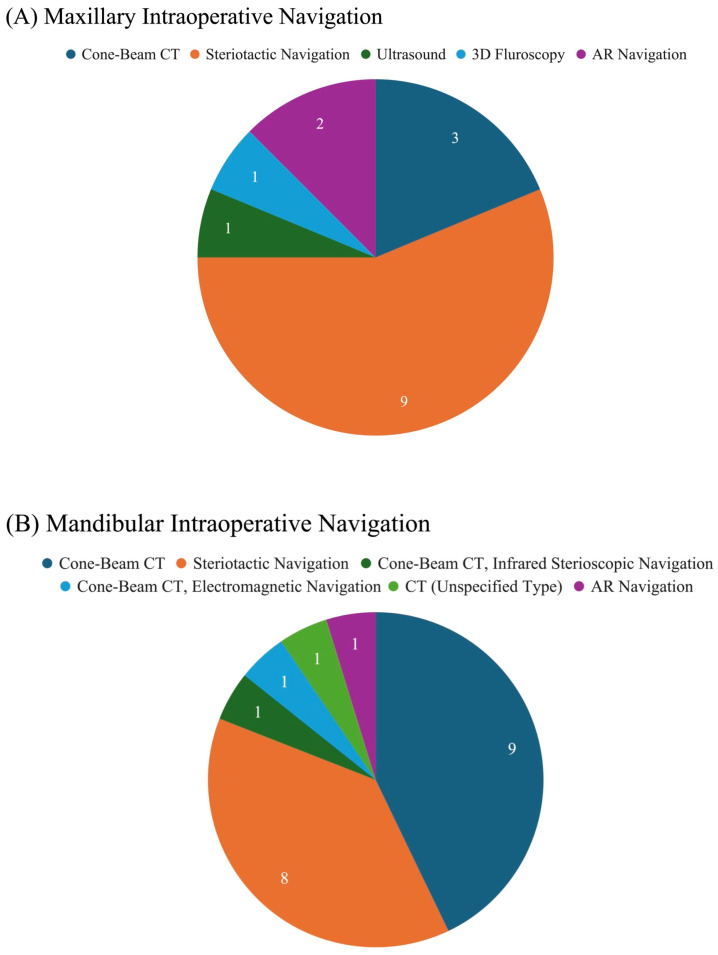
Imaging Used.

Studies reporting on maxillary surgery predominantly used stereotactic navigation (56.3%), followed by cone-beam computed tomography (CBCT) (18.8%), AR (12.5%), and ultrasound and fluoroscopy (6.3%, each) (Figure 2). CBCT was most used for ZMC and other midfacial fractures. A majority (n = 9, 56.2%) of studies investigated the clinical feasibility of the imaging technique, and the same number (56.2%) assessed if the imaging techniques increased the efficacy or quality of surgical outcomes (Table 1).

Within mandibular studies, intraoperative guidance included CBCT (n = 9, 42.9%), closely followed by stereotactic navigation (n = 8, 38.1%). There was one study (4.8%) each that used AR, CBCT combined with another electromagnetic or infrared navigation, and an unspecified CT model (Figure 2). Nine (42.9%) of the studies focused on oncologic resection or reconstruction, of which seven (77.8%) used stereotactic navigation. Seven (33.3%) investigated imaging in fracture repair, of which six (85.7%) utilized CBCT. Twelve studies (57.1%) evaluated feasibility and eleven (52.8%) assessed efficacy or quality of surgical outcomes as part of the primary aim. One investigation aimed to characterize the usage of CBCT in trauma at the study institution (Table 2) [34].

Varied metrics were used across studies to assess accuracy, prohibiting effective comparison. Only one study assessed accuracy in AR, finding a mean standard deviation of repair of 0.81 mm [24]. Accuracy of CBCT was only commented on in fracture repair. Stereotactic navigation and CBCT were associated with improvements in symmetry measures within individual studies [16,19]. In stereotactic navigation, accuracy was often assessed by comparing postoperative CT results with the virtual surgical plan, achieving mean differences under 2 mm. Few studies commented on long-term outcomes after surgery. Follow-up duration varied substantially across studies and was inconsistently reported. Where documented, follow-up, where reported, ranged from less than a week to over 10 years; however, several studies did not report follow-up duration. Longitudinal outcomes assessments were not standard across studies. Overall, imaging was safe, with minimal complications reported, although 20 (57.1%) studies did not report complication rates at all. In regard to extending the length of operative time, the utilization of intraoperative AR added approximately 60 min in one study. CBCT added approximately 1–20 min per image, and stereotactic navigation approximately 20–30 min (Table 4).

## 4. Discussion

A review of 35 articles demonstrated that intraoperative imaging in maxillofacial surgery is dominated by CBCT and stereotactic navigation. The wide variation in revisions after intraoperative CBCT (5% to 44.5% of cases, Table 4), demonstrate heterogeneous impact across surgical indications, but may also be confounded by other potential influences of revision rates, such as surgeon preference. Although AR is rapidly evolving, it was used in only 2 (5.7%) of 35 total studies; they represented 22.2% of 9 studies published in 2024 and 2025. While operative time increased across modalities, many authors commented that overall operative time may have been shortened relative to what it otherwise would have been or would shorten after a learning curve, due to the increased efficiency having imaging available for guidance. Most of the included studies aimed to establish feasibility and demonstrated that a variety of methods can be used to provide real-time feedback in the operating room. However, because these studies focused solely on surgical technique and accuracy, longitudinal complication and revision data is lacking, leaving the true long-term impact of these technologies unclear.

Comparing these results with other intraoperative imaging studies, complex facial fracture management relies heavily on CT-based imaging either intraoperatively or as a basis for surgical planning [11,12,51]. The role of CT-based imaging is also apparent in our review; 9 of 15 studies focusing on fracture repair utilized CBCT. Even when not used intraoperatively, CT was used as the foundation for intraoperative navigation and virtual surgical planning studies. Similar to the results found in this systematic review, published literature demonstrates that virtual surgical planning and intraoperative imaging are associated with improved restoration of orbital volume, zygomatic projection, safer dissection, and more accurate implant positioning [11,52,53]. Importantly, real-time verification of successful fracture reduction and implant placement enables immediate intraoperative correction, thereby reducing the need for secondary procedures and revisions, saving time and resources [52,54,55,56].

Although emerging technologies such as advanced stereotactic navigation and AR have the potential to improve intraoperative visualization, their implementation is inconsistent and often limited to implant positioning or confirmation rather than broader applications [12]. Multiple studies emphasize that, as with the slow adoption of intraoperative imaging, these technologies are constrained by technical, financial, and workflow barriers that have limited their uptake [12,52,57,58,59]. Most of the available literature remains descriptive or feasibility-focused, employing different methodologies and providing long-term data [52,56]. As a result, current evidence implies clinical benefit through improved technical accuracy rather than definitively demonstrating improvements in patient-centered outcomes, underscoring the need for large prospective, standardized studies as these technologies continue to evolve.

Complementing the more widespread use of CBCT and stereotactic navigation, one study introduced ultrasound (US) as a unique tool to assist intraoperative navigation in unilateral ZMC fractures [20]. Patients who undergo ZMC fracture repair with intraoperative US may have better outcomes such as aesthetic satisfaction and decreased need for revision surgery due to better primary reduction [51,60]. However, these studies only included small patient cohorts, and these claims need to be explored with larger patient populations. Unlike CT, US provides real-time imaging that can be used consecutively with manipulation, and may be particularly useful in managing variable-type and unstable ZMC fractures [60,61]. In initial diagnosis of craniofacial fractures, ultrasound has been shown to have similar sensitivity and specificity to CT imaging, which may support use translated to an operative setting [62]. Additionally, US minimizes the need for other intraoperative imaging, therefore reducing radiation exposure, which averages as high as 5.1–6.5 mSv with CBCT navigation [63,64]. Furthermore, US is low-cost, portable, and easily integrated into current surgical settings [65,66].

Although not included in the final dataset, a study on imaging in fibular harvesting for mandibular repair merits discussion of its novel approach and display of alternate ways in which imaging can be advantageous in mandibular repair. Cuau et al. utilized a contactless, 3D surface-scanning-based system with a structured light camera to improve navigation accuracy for fibular free flap harvesting in mandibular reconstruction [67]. This study underscores the continued development of promising technologies in mandibular reconstruction imaging that stretch beyond imaging of the face itself or traditional techniques, demonstrating that surface imaging can provide precise and consistent intraoperative navigation, for regions with minimal anatomic landmarks, such as the fibula to enhance accuracy, efficiency, and ease of intraoperative navigation.

Intraoperative imaging and navigation technologies allow surgeons to confirm fracture reduction, assess fixation accuracy, and identify subtle misalignments that would not be apparent on direct inspection alone before wound closure [68,69]. This technology is especially important in maxillofacial surgery, where even small deviations can affect function and aesthetics [12]. Prior studies have shown that facial asymmetry as small as 3 mm is perceptible to observers, underscoring the narrow margin for error acceptable in maxillofacial reconstruction [2]. Under these situations, intraoperative imaging provides objective confirmation of alignment that may complement clinical judgement and reduce the likelihood of postoperative revisions [70,71].

The clinical benefit of intraoperative imaging and navigation is likely the greatest in cases with complex or distorted anatomy such as comminuted fractures, bilateral injuries, and high-energy mechanisms such as gunshot wounds, where normal anatomic landmarks are disrupted [72]. In a retrospective case series of 337 ZMC fractures, Korner et al. found that displaced or comminuted fractures were more likely to require three or more intraoperative revisions [25]. Similar challenges exist in reconstructive settings with altered anatomy, including reconstruction following oncologic resection, or cases involving donor–recipient anatomic mismatch where preoperative plans may not fully reflect intraoperative anatomy [73,74]. Because malalignment in maxillary and mandibular surgeries can lead to visual and occlusal disruption, enhanced accuracy and symmetry repair is essential [75]. In cases of unilateral injury or reconstruction, virtual surgical planning combined with intraoperative imaging and navigation further allows restoration of symmetry, by mirroring the unaffected side and using that as a guide for anatomic alignment [22]. Notably, many studies included in this review excluded such complex cases, limiting generalizability and underestimating the true clinical value of these intraoperative technologies in patients who can benefit the most.

Despite all the potential benefits, practical considerations influence real-world adoption. The use of intraoperative imaging requires extensive training, familiarity with image interpretation, and integration into the pre-existing workflow [76]. Additionally, intraoperative imaging and navigation systems introduce technical challenges beyond conventional radiography, requiring additional experience from the surgical team for effective use [77]. Furthermore, CT-based modalities add radiation exposure and cost burdens, which must be balanced against potential benefits such as improved precision, fewer revisions, or reduced need for postoperative imaging [12,78]. Given the tradeoffs and the absence of consistent cost-effectiveness analysis data, intraoperative imaging and navigation is likely to remain a selective tool rather than a standard routine of maxillofacial surgery.

The existing evidence evaluating intraoperative imaging in maxillofacial surgery has several important limitations. Although there were some larger studies, many included studies were retrospective and involved small patient cohorts, therefore limiting statistical power and increasing susceptibility to bias [79]. In addition, many included studies lacked an appropriate comparator or control group, such as cases without using intraoperative imaging technologies, limiting the ability to attribute observed improvements directly to intraoperative imaging rather than surgeon experience, case selection, or other confounding factors. Reported endpoints were heterogeneous, with wide variations in how accuracy, symmetry, complications, and revision rates were defined and measured, limiting meaningful comparison across studies [51]. Reporting of complications and revision surgery was frequently incomplete, limiting meaningful comparison of long-term outcomes across studies or broad conclusions on clinical benefit. Importantly, no included studies evaluated cost-effectiveness of these interventions.

This review is limited by publication biases, as studies demonstrating feasibility or favorable outcomes may be more likely to be published [80]. By designing the study to focus on mandibular and maxillary imaging protocols, the study does not include other craniofacial surgery imaging technologies. Non-English language studies were excluded, and heterogeneity in study design, patient populations, imaging modalities, and outcome measurements limit the possibility of performing a meta-analysis. As a result, although current evidence supports feasibility and benefits in surgical accuracy, comparative superiority across various technologies cannot be determined.

Despite these limitations, this review provides a structured and comprehensive synthesis of current evidence on intraoperative imaging and navigation in maxillofacial surgery. By systematically evaluating surgical indications, imaging modalities, and reported outcomes, this review identifies consistent trends in surgical accuracy while also highlighting key heterogeneity in study design, comparator use, and cost-effectiveness analysis. The literature search was comprehensive and up to date, drawing from multiple large databases. These findings offer a clear guidance for future prospective studies aimed at defining the true clinical and economic value of intraoperative imaging technologies.

## 5. Conclusions

Current evidence supports the feasibility of intraoperative imaging and navigation in maxillofacial surgery. Although some studies suggest potential benefit in improving surgical precision, available data remain limited and insufficient to establish definite improvements in clinical outcomes across technologies, nor to support routine adoption across various indications. Future studies should prioritize prospective design with standardized outcome measures, encompassing accuracy of tools, complications, revision rates, and operative workflow impact. Further, cost analyses and comparative studies across technologies are essential to widespread adoption.

## Figures and Tables

**Table 1:** Characteristics of Included Maxillary Studies.

Included Maxillary Studies										
Title	Authors	Year	Country	Study Design	Total Patients (n)	Mean Age (years)	Navigation	Indication	Control Group	Postoperative Follow-Up Duration
**Cone-Beam CT Studies**										
Intraoperative imaging of zygomaticomaxillary complex fractures using a 3D C-arm system [21]	Heiland M; Schulze D; Blake F; Schmelzle R	2005	Germany	Case Series	14	43.9	Cone-Beam CT	Fracture (ZMC)	None	NR
Five-Year Experience with Routine Use of Intraoperative Cone-Beam Computed Tomography in Zygomaticomaxillary Complex Fractures [25]	Korner D; Schönegg D; Wiedemeier D; Wagner MEH; Essig H; Blumer M	2025	Switzerland	Retrospective Case Series	337	48.5	Cone-Beam CT	Fracture (ZMC)	None	At least 6 months
Symmetry-based analysis after surgical treatment of zygomaticomaxillary complex fractures using intraoperative cone-beam computed tomography: a retrospective case-control study [19]	Franke A; Matschke JB; Bučkova M; Rahrisch L; Lauer G; Leonhardt H	2025	Germany	Retrospective Case-Control	214	Study Group: 49.8 Control Group: 52.1	Cone-Beam CT	Fracture (ZMC)	Non-fracture CT (n = 82)	NR
**Steriotactic Navigation Studies**										
Navigation-guided reduction and orbital floor reconstruction in the treatment of zygomatic-orbital-maxillary complex fractures [27]	Yu H; Shen G; Wang X; Zhang S	2010	China	Case Series	6	27	Steriotactic Navigation	Fracture (Other)	None	4 weeks
Navigation-guided correction of midfacial post-traumatic deformities (Shanghai experience with 40 cases) [29]	Zhang S; Gui H; Lin Y; Shen G; Xu B	2012	China	Case Series	40	32	Steriotactic Navigation	Fracture (Mixed)	None	1 year then again at 2 years
The indication and application of computer-assisted navigation in oral and maxillofacial surgery-Shanghai’s experience based on 104 cases [28]	Yu H; Shen SG; Wang X; Zhang L; Zhang S	2013	China	Case Series	104	29 (Median)	Steriotactic Navigation	Mixed	None	5–65 months
The Use of Brainlab Navigation in Le Fort III Osteotomy [26]	Wood JS; Purzycki A; Thompson J; David LR; Argenta LC	2015	USA	Case Series	3	12	Steriotactic Navigation	Congenital	None	5–10 days
Quantitative assessment of symmetry recovery in navigation-assisted surgical reduction of zygomaticomaxillary complex fractures [16]	Bao T; Yu D; Luo Q; Wang H; Liu J; Zhu H	2019	China	Retrospective Case Series	25	40.4	Steriotactic Navigation	Fracture (ZMC)	Non-navigation surgical group	2nd week, 3rd month, 6th month, 12th month, and 18th month
Maxillectomy Reconstruction Revision Using Virtual Surgical Planning and Intraoperative Navigation [22]	Nwagu U; Swendseid B; Ross H; Ganti R; Kane A; Curry JM	2021	USA	Case Series	4	54.75	Steriotactic Navigation	Oncologic	None	NR
Improved procedure for Brown’s Class III maxillary reconstruction with composite deep circumflex iliac artery flap using computer-assisted technique [30]	Zhang WB; Soh HY; Yu Y; Guo CB; Yu GY; Peng X	2021	China	Case Report	1	27	Steriotactic Navigation	Oncologic	None	10 months
Real-Time Image-Guided Navigation in the Management of Alveolar Cleft Repair [31]	Zhang Y; Dai J; Fu X; Yang J; Fu Y; Li J	2023	China	Case Series	3	12.67	Steriotactic Navigation	Congenital	None	3 months
Adaptation of virtual surgical planning to midface reconstruction with intraoperative navigation: “navigation mediated midface reconstruction” [17]	Canter HI; Ismayilzade M; Yıldız K; Canbolat Ç; Dündar TT; Acka G; Demirak MO	2025	Turkey	Case Series	5	31.6	Steriotactic Navigation	Oncologic	None	3 months
**Augmented Reality Studies**										
Augmented Reality Navigation in Craniomaxillofacial/Head and Neck Surgery [23]	Strong EB; Patel A; Marston AP; Sadegh C; Potts J; Johnston D; Ahn D; Bryant S; Li M; Raslan O; Lucero SA; Fischer MJ; Zwienenberg M; Sharma N; Thieringer F; El Amm C; Shahlaie K; Metzger M	2025	USA, Germany, Switzerland	Retrospective Case Series	33	34.9	AR Navigation	Mixed	None	NR
Virtual surgical planning and augmented reality for fixation of plate during Le Fort I osteotomy [24]	Suenaga H; Sakakibara A; Taniguchi A; Hoshi K	2025	Japan	Case Series	6	23.2	AR Navigation	Congenital	None	NR
**Other Studies**										
Intraoperative Use of Ultrasonography in the Reduction of Zygomatico-Maxillary Complex Fractures [20]	Eswari J; Ravindran C; Deepak C	2022	India	Randomized Controlled Trial	24	29	Ultrasound	NR	Conventional reduction (blind digital palpation)	NR
On the use of intraoperative 3D-RX C-arm imaging in orthognathic surgery: a prospective non-consecutive case series study [18]	De Meurechy NKG; Decoste C; Mommaerts MY	2024	Belgium	Pospective Case Series	62	26.6	3D Fluroscopy	Mixed	None	NR

Abbreviations: AR = Augmented Reality; CT = Computed Tomography; NR = Not Reported; ZMC = Zygomaticomaxillary Complex.

**Table 2:** Characteristics of Included Mandibular Studies.

Included Mandibular Studies										
Title	Authors	Year	Country	Study Design	Total Patients (n)	Mean Age (years)	Navigation	Indication	Control Group	Postoperative Follow-Up Duration
**Cone-Beam CT Studies**										
Intraoperative 3D imaging of the facial skeleton using the SIREMOBIL Iso-C3D [33]	Heiland M; Schmelzle R; Hebecker A; Schulze D	2004	Germany	Case Series	3	28.3	Cone-Beam CT	Fracture	None	NR
Major mandibular surgical procedures as an indication for intraoperative imaging [32]	Pohlenz P; Blessmann M; Blake F; Gbara A; Schmelzle R; Heiland M	2008	Germany	Case Series	125	40.7	Cone-Beam CT	Mixed	None	NR
Clinical indication for intraoperative 3D imaging during open reduction of fractures of the neck and head of the mandibular condyle [38]	Klatt J; Heiland M; Blessmann M; Blake F; Schmelzle R; Pohlenz P	2011	Germany	Case Series	60	36.5	Cone-Beam CT	Fracture	None	NR
Clinical indication for intraoperative 3D imaging during open reduction of fractures of the mandibular angle [37]	Klatt JC; Heiland M; Marx S; Hanken H; Schmelzle R; Pohlenz P	2013	Germany	Retrospective Case Series	83	26.8	Cone-Beam CT	Fracture	None	NR
Use of Intraoperative Computed Tomography in Craniomaxillofacial Trauma Surgery [34]	Cuddy K; Khatib B; Bell RB; Cheng A; Patel A; Amundson M; Dierks EJ	2018	USA	Retrospective Case Series	161	38.9	Cone-Beam CT	Mixed	None	NR
Virtual surgical planning and cone beam computed tomography in reconstruction of head and neck tumors-pilot study [42]	Krakowczyk Ł; Piotrowska-Seweryn A; Szymczyk C; Wierzgoń J; Oleś K; Ulczok R; Donocik K; Dowgierd K; Maciejewski A	2020	Poland	Case Series	6	51	Cone-Beam CT	Oncologic	None	1 year
Use of an occlusal splint and intraoperative imaging with an intraoral approach in the management of mandibular subcondylar fractures [41]	Olivetto M; Bettoni J; Bouaoud J; Testelin S; Dakpé S; Lefranc M; Devauchelle B	2020	France	Prospective Case Series	10	33	Cone-Beam CT	Fracture	None	1 week
Evaluation of Open Reduction and Internal Fixation of Mandibular Condyle Fracture by Intraoperative Cone-Beam Computed Tomography in a Hybrid Operating Room [36]	Sukegawa S; Masui M; Kanno T; Miki M; Nakamoto H; Furuki Y	2020	Japan	Case Report	1	69	Cone-Beam CT	Fracture	None	NR
Intraoperative repositioning accuracy in transoral endoscopically-assisted mandibular subcondylar fracture repair: A 3-dimensional analysis [49]	Sakkas A; Schulze C; Scheurer M; Ebeling M; Kasper R; Schramm A; Wilde F; Schulze J	2025	Germany	Retrospective Cohort	86	37.62	Cone-Beam CT	Fracture	None	1–2 weeks
**Steriotactic Navigation Studies**										
Computer planning and intraoperative navigation for palatomaxillary and mandibular reconstruction with fibular free flaps [40]	Bell RB; Weimer KA; Dierks EJ; Buehler M; Lubek JE	2011	USA	Prospective Case Series	16	60.3	Steriotactic Navigation	Oncologic	None	4 weeks
The indication and application of computer-assisted navigation in oral and maxillofacial surgery-Shanghai’s experience based on 104 cases [28]	Yu H; Shen SG; Wang X; Zhang L; Zhang S	2013	China	Case Series	104	29 (Median)	Steriotactic Navigation	Mixed	None	5–65 months
Computer-assisted surgical planning and intraoperative navigation in the treatment of condylar osteochondroma.	Yu HB; Li B; Zhang L; Shen SG; Wang XD	2015	China	Case Series	5	25.4	Steriotactic Navigation	Oncologic	None	12–30 months
Does Intraoperative Navigation Improve the Anatomical Reduction of Intracapsular Condylar Fractures? [50]	Han C; Dilxat D; Zhang X; Li H; Chen J; Liu L	2018	China	Randomized Controlled Trial	20	28.7	Steriotactic Navigation	Fracture	Conventional open reduction (no navigation)	1, 3, 6, and 12 months
Mandibular Reconstruction with Fibula Flap and Dental Implants Through Virtual Surgical Planning and Three Different Techniques: Double-Barrel Flap, Implant Dynamic Navigation and CAD/CAM Mesh with Iliac Crest Graft [48]	Antúnez-Conde R; Salmerón JI; Díez-Montiel A; Agea M; Gascón D; Sada Á; Navarro Cuéllar I; Tousidonis M; Ochandiano S; Arenas G; Navarro Cuéllar C	2021	Spain	Retrospective Cohort	14	48.3	Steriotactic Navigation	Oncologic	None	26 months (mean; range 6–48)
Accurate transfer of bimaxillary orthognathic surgical plans using computer-aided intraoperative navigation [43]	Chen C; Sun N; Jiang C; Liu Y; Sun J	2021	China	Case Series	45	23.2	Steriotactic Navigation	Congenital	None	1 month
Expanded Transoral Microvascular Mandibular Reconstruction: A Scar-Free Approach [45]	Sun J; Li J; Lv MM; Wang L; Gupta A; Shen Y	2022	China	Case Series	9	36.1	Steriotactic Navigation	Oncologic	None	3–6 months
The role of surgical navigation in computer-assisted mandibular reconstruction: is it necessary? [46]	Soh HY; Shen SY; Yu Y; Liu S; Hu LH; Peng X; Zhang WB	2024	China	Retrospective Cohort	93	45.8	Steriotactic Navigation	Oncologic	CAD/CAM only (no navigation)	23.6 months (mean; range 3–121)
**Other Studies**										
Intraoperative cone-beam CT-guided osteotomy navigation in mandible and maxilla surgery [39]	Hasan W; Daly MJ; Chan HHL; Qiu J; Irish JC	2020	Canada	Cohort Study	5	NR	Cone-Beam CT with Infrared Sterioscopic Navigation	Oncologic	None	NR
Electromagnetic surgical navigation in patients undergoing mandibular surgery [44]	Brouwer de Koning SG; Geldof F; van Veen RLP; van Alphen MJA; Karssemakers LHE; Nijkamp J; Schreuder WH; Ruers TJM; Karakullukcu MB	2021	The Netherlands	Case Series	11	59.5	Cone-Beam CT with Electromagnetic Navigation	Oncologic	None	NR
Real-time intraoperative computed tomography can accurize virtual surgical planning on the double-barrel fibular flap for mandibular reconstruction [47]	Lin CH; Hsu CH; Adarsh K; Hsu CM; Wu CM	2022	Taiwan	Case Series	9	NR	CT (Unspecified Type)	Oncologic	None	2.3 years (mean)
Augmented Reality Navigation in Craniomaxillofacial/Head and Neck Surgery [23]	Strong EB; Patel A; Marston AP; Sadegh C; Potts J; Johnston D; Ahn D; Bryant S; Li M; Raslan O; Lucero SA; Fischer MJ; Zwienenberg M; Sharma N; Thieringer F; El Amm C; Shahlaie K; Metzger M	2025	USA, Germany, Switzerland	Case Series	33	34.9	AR Navigation	Mixed	None	NR

Abbreviations: AR = Augmented Reality; CAD/CAM = Computer-Aided Design/Computer-Aided Manufacturing; CT = Computed Tomography; NR = Not Reproted.

**Table 3:** NIH Quality Assessment Score by Study.

NIH Quality Assessment Score	
Title	Score
Accurate transfer of bimaxillary orthognathic surgical plans using computer-aided intraoperative navigation.	Fair
Adaptation of virtual surgical planning to midface reconstruction with intraoperative navigation: “navigation mediated midface reconstruction”.	Good
Augmented Reality Navigation in Craniomaxillofacial/Head and Neck Surgery.	Fair
Clinical indication for intraoperative 3D imaging during open reduction of fractures of the mandibular angle.	Fair
Clinical indication for intraoperative 3D imaging during open reduction of fractures of the neck and head of the mandibular condyle.	Fair
Computer planning and intraoperative navigation for palatomaxillary and mandibular reconstruction with fibular free flaps.	Fair
Computer-assisted surgical planning and intraoperative navigation in the treatment of condylar osteochondroma.	Fair
Does Intraoperative Navigation Improve the Anatomical Reduction of Intracapsular Condylar Fractures?	Good
Electromagnetic surgical navigation in patients undergoing mandibular surgery.	Good
Evaluation of Open Reduction and Internal Fixation of Mandibular Condyle Fracture by Intraoperative Cone-Beam Computed Tomography in a Hybrid Operating Room.	Good
Expanded Transoral Microvascular Mandibular Reconstruction: A Scar-Free Approach.	Fair
Five-Year Experience with Routine Use of Intraoperative Cone-Beam Computed Tomography in Zygomaticomaxillary Complex Fractures.	Good
Improved procedure for Brown’s Class III maxillary reconstruction with composite deep circumflex iliac artery flap using computer-assisted technique.	Good
Intraoperative 3D imaging of the facial skeleton using the SIREMOBIL Iso-C3D.	Fair
Intraoperative cone-beam CT-guided osteotomy navigation in mandible and maxilla surgery.	Good
Intraoperative imaging of zygomaticomaxillary complex fractures using a 3D C-arm system.	Fair
Intraoperative repositioning accuracy in transoral endoscopically-assisted mandibular subcondylar fracture repair: A 3-dimensional analysis.	Good
Intraoperative Use of Ultrasonography in the Reduction of Zygomatico-Maxillary Complex Fractures.	Fair
Major mandibular surgical procedures as an indication for intraoperative imaging.	Fair
Mandibular Reconstruction with Fibula Flap and Dental Implants Through Virtual Surgical Planning and Three Different Techniques: Double-Barrel Flap, Implant Dynamic Navigation and CAD/CAM Mesh with Iliac Crest Graft.	Good
Maxillectomy Reconstruction Revision Using Virtual Surgical Planning and Intraoperative Navigation.	Fair
Navigation-guided correction of midfacial post-traumatic deformities (Shanghai experience with 40 cases).	Good
Navigation-guided reduction and orbital floor reconstruction in the treatment of zygomatic-orbital-maxillary complex fractures.	Fair
On the use of intraoperative 3D-RX C-arm imaging in orthognathic surgery: a prospective non-consecutive case series study.	Good
Quantitative assessment of symmetry recovery in navigation-assisted surgical reduction of zygomaticomaxillary complex fractures.	Good
Real-Time Image-Guided Navigation in the Management of Alveolar Cleft Repair.	Good
Real-time intraoperative computed tomography can accurize virtual surgical planning on the double-barrel fibular flap for mandibular reconstruction.	Poor
Symmetry-based analysis after surgical treatment of zygomaticomaxillary complex fractures using intraoperative cone-beam computed tomography: a retrospective case-control study.	Good
The indication and application of computer-assisted navigation in oral and maxillofacial surgery-Shanghai’s experience based on 104 cases.	Good
The role of surgical navigation in computer-assisted mandibular reconstruction: is it necessary?	Good
The Use of Brainlab Navigation in Le Fort III Osteotomy.	Fair
Use of an occlusal splint and intraoperative imaging with an intraoral approach in the management of mandibular subcondylar fractures.	Good
Use of Intraoperative Computed Tomography in Craniomaxillofacial Trauma Surgery.	Good
Virtual surgical planning and augmented reality for fixation of plate during Le Fort I osteotomy.	Fair
Virtual surgical planning and cone beam computed tomography in reconstruction of head and neck tumors-pilot study.	Fair

**Table 4:** Accuracy, Safety, and Efficiency of Intraoperative Imaging.

Accuracy, Safety, and Efficiency of Intraoperative Imaging								
Navigation	Indication	Maxillary or Mandibular	Title	Revisions Due to Intraoperative Imaging	Effect on Surgical Accuracy	Complications	Secondary Reoperations	Additional Operative Time Attribued to Imaging
3D Fluroscopy	Mixed	Maxillary	On the use of intraoperative 3D-RX C-arm imaging in orthognathic surgery: a prospective non-consecutive case series study.	Segment repositioning: (n = 28) Redo-osteosynthesis (n = 13)	NR	NR	NR	NR
AR Navigation	Congenital	Maxillary	Virtual surgical planning and augmented reality for fixation of plate during Le Fort I osteotomy.	NR	Mean SD: 0.81 mm RMSD: 0.82 Error within 1 mm: 81%	NR	NR	~60 min
	Mixed	Maxillary + Mandibular	Augmented Reality Navigation in Craniomaxillofacial/Head and Neck Surgery.	NR	NR	NR	NR	NR
Cone-Beam CT	Fracture Repair	Maxillary	Five-Year Experience with Routine Use of Intraoperative Cone-Beam Computed Tomography in Zygomaticomaxillary Complex Fractures.	150/337 (44.5%)	Image quality was rated as sufficient for diagnosis in 542 scans (92%)	NR	Secondary orbital floor reconstruction with a patient-specific titanium implant: n = 25 (7.4%)	NR
	Fracture Repair	Maxillary	Intraoperative imaging of zygomaticomaxillary complex fractures using a 3D C-arm system.	NR	Authors judged image quality suitable for assessment of reduction in facial fracture	NR	None	~6 min
	Fracture Repair	Maxillary	Symmetry-based analysis after surgical treatment of zygomaticomaxillary complex fractures using intraoperative cone-beam computed tomography: a retrospective case-control study.	NR	Study Mean absolute difference: 1.5 +/− 1.3 mm Symmetry: n = 104 (78.8%) Asymmetry: n = 28 (21.2%) Control Mean absolute difference: 1.0 +/− 0.9 mm Symmetry: n = 66 (80.5%) Asymmetry: n = 16 (18.5%)	NR	NR	NR
	Fracture Repair	Mandibular	Intraoperative repositioning accuracy in transoral endoscopically-assisted mandibular subcondylar fracture repair: A 3-dimensional analysis.	NR	Mean Dice coefficient: 0.62 ± 0.18 Mean multiplanar deviation: 2.79 ± 1.53 mm Average rotational deviation was 10.18° ± 6.17°	NR	NR	NR
	Fracture Repair	Mandibular	Use of an occlusal splint and intraoperative imaging with an intraoral approach in the management of mandibular subcondylar fractures.	2/10 (20%)	NR	None	NR	NR
	Fracture Repair	Mandibular	Evaluation of Open Reduction and Internal Fixation of Mandibular Condyle Fracture by Intraoperative Cone-Beam Computed Tomography in a Hybrid Operating Room.	NR	NR	NR	NR	~8 min
	Fracture Repair	Mandibular	Clinical indication for intraoperative 3D imaging during open reduction of fractures of the mandibular angle.	4/83 (5%)	NR	NR	NR	NR
	Fracture Repair	Mandibular	Clinical indication for intraoperative 3D imaging during open reduction of fractures of the neck and head of the mandibular condyle.	4/34 (11.8%)	NR	NR	NR	~5 min
	Fracture Repair	Mandibular	Intraoperative 3D imaging of the facial skeleton using the SIREMOBIL Iso-C3D.	NR	NR	NR	NR	NR
	Mixed	Mandibular	Major mandibular surgical procedures as an indication for intraoperative imaging.	NR	NR	NR	NR	~5 min
	Mixed	Mandibular	Use of Intraoperative Computed Tomography in Craniomaxillofacial Trauma Surgery.	Overall: 28% Mandibular fracture: 13%	NR	NR	NR	~20 min
	Oncologic	Mandibular	Virtual surgical planning and cone beam computed tomography in reconstruction of head and neck tumors-pilot study.	NR	NR	RFFF necrosis (n = 1) Hemorrhage (n = 1)	Skin nectrectomy and RFFF (n = 1)	NR
Cone-Beam CT, Electromagnetic Navigation	Oncologic	Mandibular	Electromagnetic surgical navigation in patients undergoing mandibular surgery.	NR	Target Registration Error: Anatomical Landmarks: 3.2 ± 1.1 mm Cutting Guide Landmarks: 2.6 ± 1.5 mm	NR	NR	~32 min (set up)
Cone-Beam CT, Infrared Sterioscopic	Oncologic	Mandibular	Intraoperative cone-beam CT-guided osteotomy navigation in mandible and maxilla surgery.	NR	Mean cutting accuracy: <2 mm	NR	NR	NR
CT (Unspecified Type)	Oncologic	Mandibular	Real-time intraoperative computed tomography can accurize virtual surgical planning on the double-barrel fibular flap for mandibular reconstruction.	n = 6 n = 3 patients underwent revision twice	Postoperative inter-incisor midline deviation: < 2 mm (n = 5) Mean deviation at 5 landmarks (bilateral condyles, bilateral gonions, and gnathion): <1 mm	NR	None	NR
Steriotactic Navigation	Congenital	Mandibular	Accurate transfer of bimaxillary orthognathic surgical plans using computer-aided intraoperative navigation.	NR	No significant differences in preoperative plan and postoperative outcome across 12 linear and angular landmarks in 3 dimensions Mean linear difference: 0.79 mm Mean angular difference 1.20°	NR	NR	~20 min
	Congenital	Maxillary	Real-Time Image-Guided Navigation in the Management of Alveolar Cleft Repair.	NR	NR	None	NR	NR
	Congenital	Maxillary	The Use of Brainlab Navigation in Le Fort III Osteotomy.	NR	NR	None	NR	NR
	Fracture Repair	Maxillary	Navigation-guided correction of midfacial post-traumatic deformities (Shanghai experience with 40 cases).	NR	Deviation: <1 mm	None	NR	NR
	Fracture Repair	Maxillary	Quantitative assessment of symmetry recovery in navigation-assisted surgical reduction of zygomaticomaxillary complex fractures.	NR	Study: significant improvement in assymetry index (*p* < 0.05 for 4/5 landmarks) Control: significant relative difference in assymetry index (*p* < 0.05 for 3/5 landmarks)	Study: Temporary facial nerve injury n = 3 (resolved in 6 months) Control: Temporary facial nerve injury n = 2 (resolved in 6 months)	None	~60 min
	Fracture Repair	Maxillary	Navigation-guided reduction and orbital floor reconstruction in the treatment of zygomatic-orbital-maxillary complex fractures.	NR	Maximal deviation: <2 mm	None	NR	NR
	Fracture Repair	Mandibular	Does Intraoperative Navigation Improve the Anatomical Reduction of Intracapsular Condylar Fractures?	NR	Study: 0.5235 mm (range, 0.381 to 0.710 mm) Control: 1.170 mm (0.858 to 1.527 mm) *p* < 0.001	None	NR	Study: 180.4 ± 21.25 min Control: 166.8 ± 15.25 min
	Mixed	Maxillary + Mandibular	The indication and application of computer-assisted navigation in oral and maxillofacial surgery-Shanghai’s experience based on 104 cases.	NR	Mean accuracy: 1.46 ± 0.24 mm ZOMC fracture: 1.57 ± 0.29 mm TMJ ankylosis: 1.35 ± 0.17 mm Fibrous dysplasia: 1.42 ± 0.21 mm Mandibular angle hypertrophia: 1.49 ± 0.26 mm Jaw tumor 1.85 ± 0.47 mm	None	NR	NR
	Oncologic	Mandibular	The role of surgical navigation in computer-assisted mandibular reconstruction: is it necessary?	NR	Study Mean intercondylar difference: 1.28 ± 3.58 mm Mean intergonial distance: 0.11 ± 4.87 mm RMS estimates: 2.69 ± 0.41 Control Mean intercondylar difference: 2.46 ± 6.72 mm Mean intergonial distance: 2.31 ± 6.14 mm RMS estimates: 2.98 ± 1.02	Not specified by study/control group: Anastomotic complication (n = 2), Soft tissue infection (n = 1)	Not specified by study/control group: Immediate surgical re-exploration (n = 2)	NR
	Oncologic	Mandibular	Expanded Transoral Microvascular Mandibular Reconstruction: A Scar-Free Approach.	NR	NR	NR	NR	NR
	Oncologic	Mandibular	Mandibular Reconstruction with Fibula Flap and Dental Implants Through Virtual Surgical Planning and Three Different Techniques: Double-Barrel Flap, Implant Dynamic Navigation and CAD/CAM Mesh with Iliac Crest Graft.	NR	Average deviation in implant position: < 1 mm Average deviation in implant angulation: < 5°	Navigation: Implant loss in irradiated patient (n = 1) VSP Cutting Guides and Plate: Intraoral exposure of the osteosynthesis material (n = 1) VSP STL and CAD/CAM: Osseoitegration failure in irradiated patient (n = 1)	Unspecified: Revision due to partial thrombosis of anastomosed vein (n = 1)	NR
	Oncologic	Mandibular	Computer-assisted surgical planning and intraoperative navigation in the treatment of condylar osteochondroma.	NR	NR	None	NR	NR
	Oncologic	Mandibular	Computer planning and intraoperative navigation for palatomaxillary and mandibular reconstruction with fibular free flaps.	NR	NR	NR	NR	NR
	Oncologic	Maxillary	Adaptation of virtual surgical planning to midface reconstruction with intraoperative navigation: “navigation mediated midface reconstruction”.	NR	Mean difference: 0.85 mm Standard deviation: 3.55 mm	Plate exposure (n = 2)	Local flap reconstruction of exposed plates (n = 2)	NR
	Oncologic	Maxillary	Maxillectomy Reconstruction Revision Using Virtual Surgical Planning and Intraoperative Navigation.	NR	NR	None	NR	NR
	Oncologic	Maxillary	Improved procedure for Brown’s Class III maxillary reconstruction with composite deep circumflex iliac artery flap using computer-assisted technique.	NR	RMSE: 3.68 mm Average lateral segment error: 0.61 mm Average medial segment error: 0.85 mm	None	NR	NR
Ultrasound	Fracture Repair	Maxillary	Intraoperative Use of Ultrasonography in the Reduction of Zygomatico-Maxillary Complex Fractures.	NR	Study: Over-correction or over reduction: n = 0 Significanlty different measurements in all 3 planes pre- and post-reduction Control: Overcorrection: n = 4 Significanlty different measurements in 2 planes pre- and post-reduction	NR	NR	“a few extra minutes”

## Data Availability

No new data were created or analyzed in this study. Data sharing is not applicable to this article.

## Supplementary Materials

The following supporting information can be downloaded at: https://www.mdpi.com/article/10.3390/jcm15041675/s1. S1: PRISMA Checklist; S2: Mandibular Search Strategy; S3: Maxillary Search Strategy.

## References

[1] Zingg M., Laedrach K., Chen J., Chowdhury K., Vuillemin T., Sutter F., Raveh J. (1992). Classification and treatment of zygomatic fractures: A review of 1025 cases. J. Oral Maxillofac. Surg..

[2] Chu E.A., Farrag T.Y., Ishii L.E., Byrne P.J. (2011). Threshold of visual perception of facial asymmetry in a facial paralysis model. Arch. Facial Plast. Surg..

[3] Causbie J., Walters B., Lally J., Adams J., Aden J., Bevans S., Spear S., Robitschek J. (2020). Complications Following Orbital Floor Repair: Impact of Intraoperative Computed Tomography Scan and Implant Material. Facial Plast. Surg. Aesthetic Med..

[4] Nayak S.S., Gadicherla S., Roy S., Chichra M., Dhaundiyal S., Nayak V.S., Kamath V. (2023). Assessment of quality of life in patients with surgically treated maxillofacial fractures. F1000Research.

[5] Yi Y., He X., Wu Y., Wang D. (2024). Global, regional, and national burden of incidence, prevalence, and years lived with disability for facial fractures from 1990 to 2019: A systematic analysis for the Global Burden of Disease study 2019. BMC Oral Health.

[6] Bray F., Laversanne M., Sung H., Ferlay J., Siegel R.L., Soerjomataram I., Jemal A. (2024). Global cancer statistics 2022: GLOBOCAN estimates of incidence and mortality worldwide for 36 cancers in 185 countries. CA Cancer J. Clin..

[7] Salari N., Darvishi N., Heydari M., Bokaee S., Darvishi F., Mohammadi M. (2022). Global prevalence of cleft palate, cleft lip and cleft palate and lip: A comprehensive systematic review and meta-analysis. J. Stomatol. Oral Maxillofac. Surg..

[8] Sukegawa S., Kanno T., Furuki Y. (2018). Application of computer-assisted navigation systems in oral and maxillofacial surgery. Jpn. Dent. Sci. Rev..

[9] Jacobs C.A., Lin A.Y. (2017). A New Classification of Three-Dimensional Printing Technologies: Systematic Review of Three-Dimensional Printing for Patient-Specific Craniomaxillofacial Surgery. Plast. Reconstr. Surg..

[10] Shaye D.A., Tollefson T.T., Strong E.B. (2015). Use of intraoperative computed tomography for maxillofacial reconstructive surgery. JAMA Facial Plast. Surg..

[11] van Hout W.M.M.T., Van Cann E.M., Muradin M.S.M., Frank M.H., Koole R. (2014). Intraoperative imaging for the repair of zygomaticomaxillary complex fractures: A comprehensive review of the literature. J. Cranio-Maxillofac. Surg..

[12] Dubron K., Van Camp P., Jacobs R., Politis C., Shaheen E. (2022). Accuracy of virtual planning and intraoperative navigation in zygomaticomaxillary complex fractures: A systematic review. J. Stomatol. Oral Maxillofac. Surg..

[13] Page M.J., McKenzie J.E., Bossuyt P.M., Boutron I., Hoffmann T.C., Mulrow C.D., Shamseer L., Tetzlaff J.M., Akl E.A., Brennan S.E. (2021). The PRISMA 2020 statement: An updated guideline for reporting systematic reviews. BMJ.

[14] Babineau J. (2014). Product Review: Covidence (Systematic Review Software). J. Can. Health Libr. Assoc. J. Assoc. Bibl. Santé Can..

[15] Study Quality Assessment Tools|NHLBI, NIH. https://www.nhlbi.nih.gov/health-topics/study-quality-assessment-tools.

[16] Bao T., Yu D., Luo Q., Wang H., Liu J., Zhu H. (2019). Quantitative assessment of symmetry recovery in navigation-assisted surgical reduction of zygomaticomaxillary complex fractures. J. Craniomaxillofac. Surg..

[17] Canter H.I., Ismayilzade M., Yıldız K., Canbolat Ç., Dündar T.T., Acka G., Demirak M.O. (2025). Adaptation of virtual surgical planning to midface reconstruction with intraoperative navigation: “navigation mediated midface reconstruction”. Oral Surg. Oral Med. Oral Pathol. Oral Radiol..

[18] De Meurechy N.K.G., Decoste C., Mommaerts M.Y. (2024). On the use of intraoperative 3D-RX C-arm imaging in orthognathic surgery: A prospective non-consecutive case series study. Oral Maxillofac. Surg..

[19] Franke A., Matschke J.B., Bučkova M., Rahrisch L., Lauer G., Leonhardt H. (2025). Symmetry-based analysis after surgical treatment of zygomaticomaxillary complex fractures using intraoperative cone-beam computed tomography: A retrospective case-control study. Sci. Rep..

[20] Eswari J., Ravindran C., Deepak C. (2022). Intraoperative Use of Ultrasonography in the Reduction of Zygomatico-Maxillary Complex Fractures. Craniomaxillofac. Trauma Reconstr..

[21] Heiland M., Schulze D., Blake F., Schmelzle R. (2005). Intraoperative imaging of zygomaticomaxillary complex fractures using a 3D C-arm system. Int. J. Oral Maxillofac. Surg..

[22] Nwagu U., Swendseid B., Ross H., Ganti R., Kane A., Curry J.M. (2021). Maxillectomy Reconstruction Revision Using Virtual Surgical Planning and Intraoperative Navigation. Laryngoscope.

[23] Strong E.B., Patel A., Marston A.P., Sadegh C., Potts J., Johnston D., Ahn D., Bryant S., Li M., Raslan O. (2025). Augmented Reality Navigation in Craniomaxillofacial/Head and Neck Surgery. OTO Open.

[24] Suenaga H., Sakakibara A., Taniguchi A., Hoshi K. (2025). Virtual surgical planning and augmented reality for fixation of plate during Le Fort I osteotomy. Oral Maxillofac. Surg..

[25] Korner D., Schönegg D., Wiedemeier D., Wagner M.E.H., Essig H., Blumer M. (2025). Five-Year Experience with Routine Use of Intraoperative Cone-Beam Computed Tomography in Zygomaticomaxillary Complex Fractures. J. Oral Maxillofac. Surg..

[26] Wood J.S., Purzycki A., Thompson J., David L.R., Argenta L.C. (2015). The Use of Brainlab Navigation in Le Fort III Osteotomy. J. Craniofac. Surg..

[27] Yu H., Shen G., Wang X., Zhang S. (2010). Navigation-guided reduction and orbital floor reconstruction in the treatment of zygomatic-orbital-maxillary complex fractures. J. Oral Maxillofac. Surg..

[28] Yu H., Shen S.G., Wang X., Zhang L., Zhang S. (2013). The indication and application of computer-assisted navigation in oral and maxillofacial surgery-Shanghai’s experience based on 104 cases. J. Craniomaxillofac. Surg..

[29] Zhang S., Gui H., Lin Y., Shen G., Xu B. (2012). Navigation-guided correction of midfacial post-traumatic deformities (Shanghai experience with 40 cases). J. Oral Maxillofac. Surg..

[30] Zhang W.B., Soh H.Y., Yu Y., Guo C.B., Yu G.Y., Peng X. (2021). Improved procedure for Brown’s Class III maxillary reconstruction with composite deep circumflex iliac artery flap using computer-assisted technique. Comput. Assist. Surg..

[31] Zhang Y., Dai J., Fu X., Yang J., Fu Y., Li J. (2023). Real-Time Image-Guided Navigation in the Management of Alveolar Cleft Repair. Cleft Palate Craniofac. J..

[32] Pohlenz P., Blessmann M., Blake F., Gbara A., Schmelzle R., Heiland M. (2008). Major mandibular surgical procedures as an indication for intraoperative imaging. J. Oral Maxillofac. Surg..

[33] Heiland M., Schmelzle R., Hebecker A., Schulze D. (2004). Intraoperative 3D imaging of the facial skeleton using the SIREMOBIL Iso-C3D. Dentomaxillofac. Radiol..

[34] Cuddy K., Khatib B., Bell R.B., Cheng A., Patel A., Amundson M., Dierks E.J. (2018). Use of Intraoperative Computed Tomography in Craniomaxillofacial Trauma Surgery. J. Oral Maxillofac. Surg..

[35] Yu H.B., Li B., Zhang L., Shen S.G., Wang X.D. (2015). Computer-assisted surgical planning and intraoperative navigation in the treatment of condylar osteochondroma. Int. J. Oral Maxillofac. Surg..

[36] Sukegawa S., Masui M., Kanno T., Miki M., Nakamoto H., Furuki Y. (2020). Evaluation of Open Reduction and Internal Fixation of Mandibular Condyle Fracture by Intraoperative Cone-Beam Computed Tomography in a Hybrid Operating Room. J. Craniofac. Surg..

[37] Klatt J.C., Heiland M., Marx S., Hanken H., Schmelzle R., Pohlenz P. (2013). Clinical indication for intraoperative 3D imaging during open reduction of fractures of the mandibular angle. J. Craniomaxillofac. Surg..

[38] Klatt J., Heiland M., Blessmann M., Blake F., Schmelzle R., Pohlenz P. (2011). Clinical indication for intraoperative 3D imaging during open reduction of fractures of the neck and head of the mandibular condyle. J. Craniomaxillofac. Surg..

[39] Hasan W., Daly M.J., Chan H.H.L., Qiu J., Irish J.C. (2020). Intraoperative cone-beam CT-guided osteotomy navigation in mandible and maxilla surgery. Laryngoscope.

[40] Bell R.B., Weimer K.A., Dierks E.J., Buehler M., Lubek J.E. (2011). Computer planning and intraoperative navigation for palatomaxillary and mandibular reconstruction with fibular free flaps. J. Oral Maxillofac. Surg..

[41] Olivetto M., Bettoni J., Bouaoud J., Testelin S., Dakpé S., Lefranc M., Devauchelle B. (2020). Use of an occlusal splint and intraoperative imaging with an intraoral approach in the management of mandibular subcondylar fractures. J. Craniomaxillofac. Surg..

[42] Krakowczyk Ł., Piotrowska-Seweryn A., Szymczyk C., Wierzgoń J., Oleś K., Ulczok R., Donocik K., Dowgierd K., Maciejewski A. (2020). Virtual surgical planning and cone beam computed tomography in reconstruction of head and neck tumors-pilot study. Otolaryngol. Pol..

[43] Chen C., Sun N., Jiang C., Liu Y., Sun J. (2021). Accurate transfer of bimaxillary orthognathic surgical plans using computer-aided intraoperative navigation. Korean J. Orthod..

[44] Brouwer de Koning S.G., Geldof F., van Veen R.L.P., van Alphen M.J.A., Karssemakers L.H.E., Nijkamp J., Schreuder W.H., Ruers T.J.M., Karakullukcu M.B. (2021). Electromagnetic surgical navigation in patients undergoing mandibular surgery. Sci. Rep..

[45] Sun J., Li J., Lv M.M., Wang L., Gupta A., Shen Y. (2022). Expanded Transoral Microvascular Mandibular Reconstruction: A Scar-Free Approach. J. Oral Maxillofac. Surg..

[46] Soh H.Y., Shen S.Y., Yu Y., Liu S., Hu L., Peng X., Zhang W. (2024). The role of surgical navigation in computer-assisted mandibular reconstruction: Is it necessary?. Med. Oral Patol. Oral Cir. Bucal.

[47] Lin C.H., Hsu C.H., Adarsh K., Hsu C.M., Wu C.M. (2022). Real-time intraoperative computed tomography can accurize virtual surgical planning on the double-barrel fibular flap for mandibular reconstruction. J. Plast. Reconstr. Aesthetic Surg..

[48] Antúnez-Conde R., Salmerón J.I., Díez-Montiel A., Agea M., Gascón D., Sada Á., Cuéllar I.N., Tousidonis M., Ochandiano S., Arenas G. (2021). Mandibular Reconstruction with Fibula Flap and Dental Implants Through Virtual Surgical Planning and Three Different Techniques: Double-Barrel Flap, Implant Dynamic Navigation and CAD/CAM Mesh with Iliac Crest Graft. Front. Oncol..

[49] Sakkas A., Schulze C., Scheurer M., Ebeling M., Kasper R., Schramm A., Wilde F., Schulze J. (2025). Intraoperative repositioning accuracy in transoral endoscopically-assisted mandibular subcondylar fracture repair: A 3-dimensional analysis. J. Stomatol. Oral Maxillofac. Surg..

[50] Han C., Dilxat D., Zhang X., Li H., Chen J., Liu L. (2018). Does Intraoperative Navigation Improve the Anatomical Reduction of Intracapsular Condylar Fractures?. J. Oral Maxillofac. Surg..

[51] Gong J., Zhao R., Zhang W., Li J., Yuan Z., Ma D. (2023). Role of Intraoperative Imaging in Improving Closed Reduction of Zygomatic Arch Fractures: A Systematic Review and Meta-analysis. J. Oral Maxillofac. Surg..

[52] Austin R.E., Antonyshyn O.M. (2012). Current Applications of 3-D Intraoperative Navigation in Craniomaxillofacial Surgery: A Retrospective Clinical Review. Ann. Plast. Surg..

[53] Longeac M., Depeyre A., Pereira B., Barthelemy I., Pham Dang N. (2021). Virtual surgical planning and three-dimensional printing for the treatment of comminuted zygomaticomaxillary complex fracture. J. Stomatol. Oral Maxillofac. Surg..

[54] DeLong M.R., Gandolfi B.M., Barr M.L., Datta N., Willson T.D., Jarrahy R. (2019). Intraoperative Image-Guided Navigation in Craniofacial Surgery: Review and Grading of the Current Literature. J. Craniofac. Surg..

[55] Sritharan R., Arya R., Abdelrahman A., Parmar S., Sharp I., Breeze J. (2023). Justifying the implementation of intraoperative computed tomography for midface fracture treatment in improving outcomes. Br. J. Oral Maxillofac. Surg..

[56] Liu Y., Enin K., Sciegienka S., Hardi A., Spataro E. (2023). Intraoperative Computed Tomography Use in Orbital Fracture Repair: A Systematic Review and Meta-Analysis. Facial Plast. Surg. Aesthetic Med..

[57] Chen G., Zeng W., Yin H., Yu Y., Ju R., Tang W. (2020). The Preliminary Application of Augmented Reality in Unilateral Orbitozygomatic Maxillary Complex Fractures Treatment. J. Craniofac. Surg..

[58] Guo Z., Leong M.C.W., Su H., Kwok K.W., Chan D.T.M., Poon W.S. (2018). Techniques for Stereotactic Neurosurgery: Beyond the Frame, Toward the Intraoperative Magnetic Resonance Imaging–Guided and Robot-Assisted Approaches. World Neurosurg..

[59] Walker T.L.J., Bamford R., Finch-Jones M. (2017). Intraoperative ultrasound for the colorectal surgeon: Current trends and barriers. ANZ J. Surg..

[60] Buller J., Zirk M., Kreppel M., Maus V., Zöller J.E. (2019). Intraoperative Ultrasound Control of Zygomatic Arch Fractures: Does Additional Imaging Improve Reduction Quality?. J. Oral Maxillofac. Surg..

[61] Sasaki K., Sasaki M., Oshima J., Aihara Y., Shibuya Y., Sekido M. (2024). Transmalar Kirshner Wire Fixation Under Ultrasound Scanning is Useful for Unstable Zygomatic Arch Fracture Combined with Zygomatic Body Fracture. J. Craniofac. Surg..

[62] Adeyemo W.L., Akadiri O.A. (2011). A systematic review of the diagnostic role of ultrasonography in maxillofacial fractures. Int. J. Oral Maxillofac. Surg..

[63] Tu T.H., Kuo Y.H., Chang C.C., Kuo C.-H., Chang H.-K., Fay L.-Y., Yeh M.-Y., Ko C.-C., Huang W.-C., Wu J.-C. (2023). Comparison of intraoperative cone-beam CT versus preoperative fan-beam CT for navigated spine surgery: A prospective randomized study. J. Neurosurg. Spine.

[64] Costa F., Tosi G., Attuati L., Cardia A., Ortolina A., Grimaldi M., Galbusera F., Fornari M. (2016). Radiation exposure in spine surgery using an image-guided system based on intraoperative cone-beam computed tomography: Analysis of 107 consecutive cases. J. Neurosurg. Spine.

[65] Zaffino P., Moccia S., De Momi E., Spadea M.F. (2020). A Review on Advances in Intra-operative Imaging for Surgery and Therapy: Imagining the Operating Room of the Future. Ann. Biomed. Eng..

[66] Machi J., Oishi A.J., Furumoto N.L., Oishi R.H. (2004). Intraoperative ultrasound. Surg. Clin. N. Am..

[67] Cuau L., De Boutray M., Cavalcanti Santos J., Zemiti N., Poignet P. (2023). Contactless surface registration of featureless anatomy using structured light camera: Application to fibula navigation in mandible reconstruction. Int. J. Comput. Assist. Radiol. Surg..

[68] Gong J., Zhang W., Zhao R., Zhang W., Wang B., Ma D. (2023). The Role of Intraoperative Navigation in Surgical Treatment of Unilateral Zygomatic Complex Fractures: A Systematic Review and Meta-Analysis. J. Oral Maxillofac. Surg. Off. J. Am. Assoc. Oral Maxillofac. Surg..

[69] Sharma P., Rattan V., Rai S., Chhabbra R. (2021). Does Intraoperative Computed Tomography Improve the Outcome in Zygomatico-Orbital Complex Fracture Reduction?. J. Maxillofac. Oral Surg..

[70] Alkhayatt N.M., Alzahrani H.H., Ahmed S., Alotaibi B.M., Alsaggaf R.M., Alalmuaysh A.M., Alomair A.A. (2024). Computer-assisted navigation in oral and maxillofacial surgery: A systematic review. Saudi Dent. J..

[71] Singh M., Ricci J.A., Caterson E.J. (2015). Use of Intraoperative Computed Tomography for Revisional Procedures in Patients with Complex Maxillofacial Trauma. Plast. Reconstr. Surg. Glob. Open.

[72] Shalabi M.M., Darwich K.M.A., Kheshfeh M.N., Hajeer M.Y. (2025). Efficiency and Applicability of Virtual Surgical Planning in Maxillofacial and Mandibular Bone Reconstruction: A Narrative Review. Clin. Pract..

[73] Udhay P., Bhattacharjee K., Ananthnarayanan P., Sundar G. (2019). Computer-assisted navigation in orbitofacial surgery. Indian J. Ophthalmol..

[74] Anand M., Panwar S. (2021). Role of Navigation in Oral and Maxillofacial Surgery: A Surgeon’s Perspectives. Clin. Cosmet. Investig. Dent..

[75] Kim S.Y., Choi Y.H., Kim Y.K. (2018). Postoperative malocclusion after maxillofacial fracture management: A retrospective case study. Maxillofac. Plast. Reconstr. Surg..

[76] Nasir N., Cercenelli L., Tarsitano A., Marcelli E. (2023). Augmented reality for orthopedic and maxillofacial oncological surgery: A systematic review focusing on both clinical and technical aspects. Front. Bioeng. Biotechnol..

[77] Kang D.H. (2024). Intraoperative navigation in craniofacial surgery. Arch. Craniofacial Surg..

[78] Barrett D.M., Halbert T.W., Fiorillo C.E., Park S.S., Christophel J.J. (2015). Cost-based decision analysis of postreduction imaging in the management of mandibular fractures. JAMA Facial Plast. Surg..

[79] Tipton E., Hallberg K., Hedges L.V., Chan W. (2017). Implications of Small Samples for Generalization: Adjustments and Rules of Thumb. Eval. Rev..

[80] Canestaro W.J., Hendrix N., Bansal A., Sullivan S.D., Devine E.B., Carlson J.J. (2017). Favorable and publicly funded studies are more likely to be published: A systematic review and meta-analysis. J. Clin. Epidemiol..

