# Supplementary material for: The Current State of Intraoperative Imaging in Maxillofacial Surgery: A Systematic Review
Source: J Clin Med. 2026 Feb 23;15(4):1675. doi: 10.3390/jcm15041675 (PMC12941712; doi:10.3390/jcm15041675)
Supplement: Supplementary file 1 [file jcm-15-01675-s001.zip › Search Strategy_Maxilla.pdf]

```
(
  "Maxilla"[MeSH Terms] OR "Maxilla"[Majr]
  OR maxilla[tiab] OR maxillary[tiab] OR maxillas[tiab]
)
AND
(
  "Three-Dimensional Imaging"[MeSH Terms]
  OR "Imaging, Three-Dimensional"[MeSH Terms]
  OR "Computer-Aided Design"[MeSH Terms]
  OR "Printing, Three-Dimensional"[MeSH Terms]
  OR "Surgical Planning"[MeSH Terms]
  OR "Virtual Surgical Planning"[tiab]
  OR "3D planning"[tiab]
  OR "three dimensional"[tiab]
  OR "3D printed"[tiab]
  OR "3D printing"[tiab]
  OR "computer assisted design"[tiab]
  OR "computer aided design"[tiab]
  OR "computer-assisted surgical planning"[tiab]
  OR "virtual modeling"[tiab]
  OR "virtual plan"[tiab]
  OR "CAD/CAM"[tiab]
```
